# Anti-nuclear antibody and a granuloma could be biomarkers for iCIs-related hepatitis by anti-PD-1 treatment

**DOI:** 10.1038/s41598-022-07770-8

**Published:** 2022-03-07

**Authors:** Yasuteru Kondo, Junichi Akahira, Tatsuki Morosawa, Yukihiro Toi, Akashi Endo, Hiroaki Satio, Mareyuki Endo, Shunichi Sugawara, Yasuhito Tanaka

**Affiliations:** 1 Department of Hepatology, Sendai Kousei Hospital, 4-15 Hirose-machi, Aoba-ku, Sendai City, Miyagi 980-0873 Japan; 2 Department of Pathology, Sendai Kousei Hospital, 4-15 Hirose-machi, Aoba-ku, Sendai City, Miyagi 980-0873 Japan; 3 Department of Pulmonary Medicine, Sendai Kousei Hospital, 4-15 Hirose-machi, Aoba-ku, Sendai City, Miyagi 980-0873 Japan; 4 Department of Gastroenterology, Sendai Kousei Hospital, 4-15 Hirose-machi, Aoba-ku, Sendai City, Miyagi 980-0873 Japan; 5 Department of Gastroenterology and Hepatology, Faculty of Life Sciences, Kumamoto University, 1-1-1 Honjo, Chuo-ku, Kumamoto, 860-8556 Japan

**Keywords:** Immunotherapy, Tumour immunology

## Abstract

It has been reported that various kinds of immune checkpoint inhibitors (iCIs) could induce immune-related liver damage. We should focus on the programmed cell death-receptor-1 (PD-1) antibody and non-small cell lung cancer (NSCLC) to analyze the characteristics of hepatitis related to iCIs and find factors that could be useful biomarkers for the diagnosis. A single-center retrospective study of 252 NSCLC patients who received PD-1 antibody (nivolumab or pembrolizumab). Some of the biochemical markers and immunological markers were analyzed during PD-1-antibody treatment with or without ALT elevation. Histopathological features were reviewed by a single expert of hepatic pathology focusing on the following features: fibrosis, portal inflammation, lobular inflammation, lobular necrosis. The formation of macro- and micro-granulomas was also evaluated. The frequency of liver damage induced by nivolumab including grade 1 to 4 (ALT) was 41.9% (78/186 patients). The positive rate of anti-nuclear antibody in the nivolumab group with iCIs-related hepatitis was significantly higher than that in the nivolumab group without iCIs-related hepatitis (*p* = 0.00112). Granulomatous changes were significantly increased in patients with iCIs-related hepatitis compared with DILI and AIH patients (*p* < 0.05). The ratios of inflammatory cells CD4/CD8, and CD138/CD3 in ICIs-related hepatitis were significantly lower than those in AIH or DILI patients (*p* < 0.05). We demonstrated that the pre-existing ANA and characteristic liver histology including CD8^+^ cells dominancy and granulomatous hepatitis could be biomarkers for the diagnosis of iCIs-related hepatitis in the NSCLC with anti-PD-1 therapy.

## Introduction

Various kinds of Immune-check point inhibitors (iCIs) that target immune-suppressive molecules to enhance cytotoxic T lymphocytes (CTLs) could improve the survival in patients with advanced non-small cell lung cancer. Among them, anti-programmed cell death protein 1 (anti-PD-1) is widely used. However, it has been reported that various kinds of immune-related adverse events (irAEs) might be induced by dysregulating the immune system. The Department of Pulmonary Medicine in our institute has reported various kinds of irAEs in advanced non-small cell lung cancers patients treated with anti-PD-1^1^.

It has been reported that the most common phenotype of iCIs-related liver damage was iCIs-related hepatitis^2^. However, the detailed characteristics of iCIs-related hepatitis induced by treatment with anti-PD-1 have not been clarified. Some groups described that a characteristic liver histology could be observed in patients with liver injury during the treatment with iCIs^3–5^. An analysis of immune cell subsets such as CD8 positive cells and CD4 positive cells was carried out in iCIs-related liver injury^6,7^. However, the characteristics of liver histopathology and the subsets of immune cells from iCIs-related hepatitis have not been clarified due to the limited number of samples.

In addition to the histopathology of iCIs-related hepatitis, we should consider possible biomarkers that might predict hepatitis after using iCIs. It has been reported that auto-immune disorders such as paraneoplastic syndromes could be potential biomarkers for predicting immune-related liver injury induced by treatment with iCIs^8^. Immune-related liver diseases such as auto-immune hepatitis (AIH) and primary biliary cholangitis (PBC) could be exacerbated by using iCIs. Therefore, an analysis of potential biomarkers to predict the onset of AIH or PBC such as anti-nuclear antibody (ANA) and anti-mitochondria antibody (AMA) might be useful for preventing liver injury induced by iCIs. Some groups including ours have reported that preexisting antibodies and rheumatoid factor were common among patients who developed irAEs^1,9^. However, the relation between preexisting antibodies and immune-related liver injury induced by iCIs has not been clarified. In this study, we tried to identify possible biomarkers to detect iCIs-related hepatitis induced by PD-1 antibody in lung cancer patients by analyzing the histopathology of liver and serum markers.

## Materials and methods

### Study design and inclusion criteria

This study was approved by the Ethics Committee of Sendai Kousei Hospital following the ethical guidelines of the 1975 Declaration of Helsinki, and written informed consent was obtained from each individual.. Patients with advanced non-small cell lung cancer (NSCLC) who were treated with anti-PD-1 [nivolumab (3 mg/kg every 2 weeks) (186 patients) or pembrolizumab (200 mg every 3 weeks) (66 patients)] monotherapy at Sendai Kousei Hospital between January 2016 and June 2018 were enrolled in this study. We focused on the patients treated with monotherapy of PD-1 antibody (nivolumab or pembrolizumab) to exclude other factors potentially inducing liver damage such as molecular targeting agents, other iCIs and cell-killing anticancer drugs. In addition to the regimen of treatment, we focused on NSCLC patients to exclude the effects of primary cancer influencing the immune reactions.

### Biochemical analysis and clinical factors analysis

Patients with iCIs-related hepatitis were diagnosed by the following criteria. Alanine aminotransferase (ALT) elevations were consistent at least two weeks after using PD-1 antibody. The ALT elevations were dominant compared with aspartate aminotransferase (AST) and alkaline phosphatase (ALP) elevations. The patients were excluded if they had liver metastasis, disseminated intravascular coagulation, multiple organ failure, alcohol drinking (> 30 g/day), chronic hepatitis C. All HBsAg positive patients (3 patients) were inactive carriers. Age, sex and tissue type of lung cancer were compared between patients with iCIs-related hepatitis and those without iCIs-related hepatitis. The grade of liver damage was analyzed using common terminology criteria for adverse events (CTCAE) v5.0. We evaluated iCIs-related hepatitis together with ALT elevation using the grading of CTCAE v5.0. Some of the biochemical markers and immunological markers were analyzed during PD-1-antibody treatment with or without iCIs-related hepatitis. Overall survival with or without iCIs-related hepatitis was analyzed using the Kaplan–Meier method.

### Histological evaluation

Liver biopsies were carried out immediately after consulting the Department of Hepatology about liver damage in some patients treated with PD-1 antibody. The selection of patients who should be undergo liver biopsy was evaluated by two hepatologists to exclude the risk of bleeding and the progression of lung cancer. All biopsy specimens were fixed in formalin and paraffin embedded (FFPE). Sections (3 µm) were then stained with hematoxylin and eosin. Histopathological features were reviewed by a single expert of hepatic pathology focusing on the following features: fibrosis according to Brunt’s criteria (G1–G4)^10^, portal inflammation (0–3), lobular inflammation (0–3), lobular necrosis (0–3). The formation of macro- and micro-granulomas was also evaluated.

### Immunohistochemical evaluation

Immunohistochemical staining for T-cell markers (CD3, CD4, CD8), B-cell marker (CD20), and plasma cell marker (CD138) was performed using an automatic immunostainer (Ventana BenchMark GX, Roche). The antibodies and sources were as follows: CD3 (mouse monoclonal, Leica, Germany), CD4 (mouse monoclonal, Nichirei, Japan), CD8 (mouse monoclonal, DAKO, Denmark), CD20 (mouse monoclonal, DAKO, Denmark) and CD138 (mouse monoclonal, Nichirei, Japan). Immunohistochemical analyses were performed in all of the patients and compared to those with typical auto-immune hepatitis (AIH) (n = 3) and classical drug-induced liver injury (DILI) (n = 3). We also performed double-staining immunohistochemistry, using DAB and fast-red staining, to visualize the proportion of CD4 and CD8 positive lymphocytes in case 17.

### Statistical analysis

**Figure 1:**
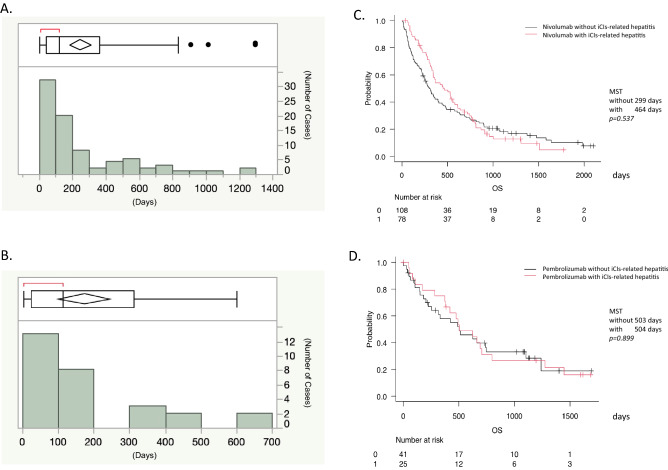
The median onset time points of iCIs-related hepatitis induced by the administration of nivolumab and pembrolizumab are shown (**A**) nivolumab and (**B**) pembrolizumab). X-axis shows the onset time points of iCIs-related hepatitis. Y-axis shows the number of patients with iCIs-related hepatitis. The survival curves of lung cancer patients with or without iCIs-relate hepatitis; nivolumab (**C**) and pembrolizumab (**D**) are shown. X-axis shows the survival days after the start of PD-1 antibody treatment. Y-axis shows the probability among the included patients.

The survival time was calculated using the Kaplan–Meier method and the analysis of log-rank test (Fig. 1C,D). χ^2^ test or *independent t test* was employed to compare the differences between the patients treated by nivolumab without liver damage and those treated by nivolumab with liver damage using JMP Pro 15.0 (Table 1). We also compared the clinical factors of irAEs induced by pembrolizumab with liver damage or without liver damage in the same way (Table 2). We analyzed six factors (age, sex, anti-nuclear antibody, anti-mitochondria antibody, IgG and IgM) to identify the predictive risk factors for iCIs-related hepatitis by multivariate analysis (Table 3). Multivariate analysis was carried out by logistic regression analysis (Table 3). We also compared the clinical factors of irAEs induced by pembrolizumab with liver damage or without liver damage in the same way. *Mann–Whitney U test* was employed to compare the frequencies of immune cell subsets between the patients treated with PD-1 antibody and control subjects (Table 4).

**Table 1:** Patients’ characteristics treated by nivolumab.

	Nivolumab without iCIs-related hepatitis	Nivolumab with iCIs-related hepatitis	Univariate analysis
	(Without ALT elevation)	(With ALT elevation)	
Subjects	108	78	n.a
Age (average)	69.9	68.9	n.s
Sex (male/female)	(82/26)	(65/13)	n.s
Type of lung cancer (adenocarcinoma/squemas cell carcinoma/other)	(64/39/5)	(42/33/3)	n.s
Liver damage (G1/G2/G3/G4)	NA	(55/11/10/2)	n.a
Peak T-bil average (SD)	1.1 (1.4)	2.0 (3.4)	*p* = 0.0410
Peak ALT average (SD)	34.8 (25.0)	228.4 (443.5)	*p* < 0.0001
Peak ALP average (SD)	501 (390.7)	869 (855.8)	*p* = 0.0015
Peak LDH average (SD)	575.3 (757.3)	842.1 (1251.9)	*p* = 0.0940
Treatments for liver damage (observation/SNMC/steroid/steroid-pulse/steroid + azatiopline)	NA	(63/5/4/0/3)	n.a
HBsAg (positive/negative)	(1/107)	(1/77)	n.s
Anti-nuclear antibody (positive/negative)	(22/86)	(29/49)	*p* < 0.0112
Anti-mitochondria antibody (positive/negative)	(3/105)	(2/76)	n.s
IgG average (SD)	1362 (475)	1424 (509)	n.s
IgM average (SD)	83.5 (45.7)	87.6 (51.5)	n.s

*SD* Standard deviation.

**Table 2:** Patients’ characteristics treated by pembrolizumab.

	Pembrolizumab without iCIs-related hepatitis	Pembrolizumab with iCIs-related hepatitis	Univariate analysis
	(Without ALT elevation)	(With ALT elevation)	
Subjects	41	25	n.a
Age (average)	72.1	64.6	*p* = 0.0121
Sex (male/female)	(31/10)	(22/3)	*p* = 0.2195
Type of lung cancer (Adenocarcinoma/Squemas cell Carcinoma/Other)	(16/19/6)	(18/7/0)	n.s
Liver Damage (G1/G2/G3/G4)	NA	(17/3/2/3)	n.a
Peak T-bil average (SD)	1.1 (1.0)	1.8 (2.0)	*p* = 0.1372
Peak ALT average (SD)	42.6 (49.7)	485.7 (1267)	*p* = 0.0043
Peak ALP average (SD)	499 (463.6)	864 (908.7)	*p* = 0.0655
Peak LDH average (SD)	380.2 (309.3)	579.8 (598.9)	*p* = 0.1299
Treatments for liver damage (observation/SNMC/steroid/steroid-pulse/steroid + azatiopline)	NA	(22/1/1/1/0)	n.a
HBsAg (positive/negatige)	(1/40)	(0/25)	n.s
Anti-nuclear antibody (positive/negative)	(5/36)	(12/13)	*p* = 0.0013
Anti-mitochondria antibody (positive/negative)	(1/40)	(0/25)	n.s
IgG (SD)	1516 (464)	1572 (658)	n.s
IgM (SD)	86.6 (42.2)	93.4 (33.8)	n.s

*SD* Standard deviation.

**Table 3:** Possible risk factors of iCIs-related hepatitis.

	Nivoloumab without iCIs-related hepatitis	Nivolumab with iCIs-related hepatitis	Univariate analysis	Multivariate analysis		
	(Without ALT elevation)	(With ALT elevation)	*p* value	HR	95%CI	*p* value
Age (average)	69.9	68.9	n.s	0.997	0.962–1.033	*p* = 0.8550
Sex (male/female)	(82/26)	(65/13)	n.s	1.504	0.691–3.271	*p* = 0.3037
Anti-nuclear antibody (positive/negative)	(22/86)	(29/49)	*p* = 0.0112	2.133	1.085–4.194	*p* = 0.0281
Anti-mitochondria antibody (positive/negative)	(3/105)	(2/76)	n.s	1.198	0.181–7.925	*p* = 0.8513
IgG average (SD)	1362 (475)	1424 (509)	n.s	1	0.999–1.000	*p* = 0.7687
IgM average (SD)	83.5 (45.7)	87.6 (51.5)	n.s	1	0.994–1.007	*p* = 0.9011

	Pembrolizumab without iCIs-related hepatitis	Pembrolizumab with iCIs-related hepatitis	Univariate analysis	Multivariate analysis		
	(Without ALT elevation)	(With ALT elevation)	*p* value	HR	95%CI	*p* value
Age (average)	72.1	64.6	*p* = 0.0121	0.896	0.829–0.967	*p* = 0.0050
Sex (male/female)	(31/10)	(22/3)	n.s	2.332	0.312–17.42	*p* = 0.4091
Anti-nuclear antibody (positive/negative)	(5/36)	(12/13)	*p* = 0.0013	7.834	1.743–35.21	*p* = 0.0073
Anti-mitochondria antibody (positive/negative/unknown)	(1/40)	(0/25)	n.s	n.s	n.s	*p* = 0.9998
IgG (SD)	1516 (463.5)	1572 (658.4)	n.s	1	0.999–1.001	*p* = 0.8654
IgM (SD)	86.6 (42.2)	93.4 (33.8)	n.s	0.986	0.963–1.008	*p* = 0.2166

*SD* Standard deviation.

**Table 4:** Clinicopathological features.

Case	Age/sex	Drug	Clinical grading	Portal inflammation	lobular inflammation	Necrosis	Fibrosis	B/T ratio (CD20/CD3)	CD4/CD8 ratio	CD138/CD3 ratio	Micro-granuloma	Macro-granuloma
1	69/M	Nivolumab	G1	0	1	1	1	0.016	0.96	0.051	+	
2	63/M	Nivolumab	G1	0	1	1	1	0.1	1.5	0.025	+	
3	62/M	Nivolumab	G1	1	1	1	1	0.057	2.12	0	+	
4	72/M	Nivolumab	G1	1	1	1	1	0	0.27	0.079		
5	31/F	Nivolumab	G1	0	1	0	1	0	0.27	0		
6	63/F	Nivolumab	G1	1	2	1	1	0.032	0.94	0.048	+	
7	69/M	Pembrolizumab	G1	1	2	2	1	0.018	0.1	0	+	
8	63/M	Pembrolizumab	G2	1	2	1	1	0	2.2	0.063	+	
9	74/M	Pembrolizumab	G2	2	1	1	1	0.089	0.51	0.071	+	
10	72/M	Pembrolizumab	G2	2	1	1	2	0.047	0.26	0.075	+	
11	73 M	Pembrolizumab	G2	1	1	1	1	0	0.73	0.031	+	
12	79/F	Nivolumab	G3	2	2	2	2	0.027	0.83	0.04	+	
13	88/F	Nivolumab	G3	3	3	2	2	0.029	0.56	0.086	++	
14	59/M	Nivolumab	G3	2	2	1	1	0	0.11	0	+	
15	73/M	Pembrolizumab	G3	2	3	2	3	0.07	0.16	0.078		+
16	57/M	Pembrolizumab	G3	2	3	3	3	0.023	0.71	0.023		+
17	77/M	Nivolumab	G4	3	3	3	4	0.11	0.85	0.087		+
18	76/F	Nivolumab	G4	3	3	3	3	0	0.062	0.036	+	
							Median	0.024	0.63	0.044		
19	68/M	AIH		3	2	2	2	0.053	2.45	0.21	+	
20	67/F	AIH		2	2	2	2	0.21	1.89	0.41		
21	41/F	AIH		3	3	2	3	0.094	1	0.083		
							Median	0.094	1.89	0.2		
22	79/M	DILI		2	3	2	3	0.19	0.9	0.64		
23	68/F	DILI		2	3	2	2	0.2	1.26	0.4		
24	57/F	DILI		3	3	3	2	0.06	1.08	0.17		
							Median	0.2	1.08	0.4		

## Results

### Characteristics of iCIs related hepatitis induced by anti-PD-1 antibody

The median onset time points of iCIs-related hepatitis induced by the administration of nivolumab and pembrolizumab were 119 days and 114 days, respectively (Fig. 1A,B). However, the iCIs-related hepatitis induced by the administration of PD-1 antibody occurred at various time points. The peak total bilirubin, ALT and ALP levels with liver damage induced by PD-1 antibody (nivolumab and pembrolizumab) administration are shown in Tables 1 and 2. The frequency of liver damage induced by nibolumab including grade 1 to 4 (ALT) was 41.9% (78/186 patients). The numbers of patients with grade 1/2/3/4 liver damage (ALT) were 55/11/10/2 patients, respectively. A HBsAg positive patient (inactive carrier) had a grade 1 ALT elevation. However, no significant decline of HBsAg had been detect in these patients. The frequencies of iCIs-related hepatitis induced by pembrolizumab including grade 1 to 4 (ALT) were 37.9% (25/66). The numbers of patients with grade 1/2/3/4 liver damage (ALT) were 17/3/2/3 patients, respectively.

Most of the patients with iCIs-related hepatitis induced by PD-1 antibody recovered by observation or strong neo minophagen C® (SNMC) consisting of monoammonium glycyrrhizinate, glycine, aminoacetic acid and L-Cystein hydrochloride hydrate administration. However, some of the patients needed steroid or steroid with azathioprine. We could not determine the cell type of lung cancer that could easily induce liver damage by the administration of PD-1 antibody (Tables 1 or 2).

The overall survival with or without iCIs related hepatitis was analyzed using the Kaplan–Meier method and the analysis of log-rank test. The survival curves of HCC patients with or without iCIs-related hepatitis after treatment of nivolumab were almost the same (Fig. 1C). The median survival time (MST) of lung cancer patients with iCIs (nivolumab)-related hepatitis(−)/iCIs-related hepatitis(+) was 299 days/464 days (*p* = 0.537). The survival curves of HCC patients with or without iCIs-related hepatitis after treatment of pembrolizumab were almost the same (Fig. 1D). The MST of lung cancer patients with iCIs (pembrolizumab)-related hepatitis(−)/iCIs-related hepatitis(+) was 503 days/504 days (*p* = 0.899).

### Possible serum biomarkers for iCIs-related hepatitis induced by anti-PD-1 antibody

We analyzed some immunological factors (age, sex, anti-nuclear antibody and anti-mitochondrial antibody, IgG and IgM) to predict the susceptibility to liver damage induced by PD-1 antibody (Table 3). The positive rate of anti-nuclear antibody in the nivolumab with iCIs-related hepatitis group was significantly higher than that in the nivolumab without iCIs-related hepatitis group by using univariate analysis (*p* = 0.0112). Moroever, we could determine a significant difference in the positive rate of anti-nuclear antibody and age between pembrolizumab with iCIs-related hepatitis and that without iCIs-related hepatitis (*p* = 0.0013 and 0.0121). In addition to the univariate analysis, multivariate analysis was carried out to detect the possible risk factors for iCIs-related hepatitis before the PD-1 treatment. The positivity of anti-nuclear antibody could be detected as a risk factor for iCIs-related hepatitis among the patients treated with nivolumab by multivariate analysis (hazard ratio 2.133: 95%CI 1.085–4.194: *p* = 0.0281). In addition, the positivity of anti-nuclear antibody and age could be detected as the risk factors for iCIs-related hepatitis among the patients treated with pembrolizumab by multivariate analysis (hazard ratio 7.834: 95%CI 1.743–35.21: *p* = 0.0073 and hazard ratio 0.896: 95%CI 0.896: *p* = 0.0050, *respectively*).

### Liver histology of hepatitis induced by PD-1 antibody

**Figure 2:**
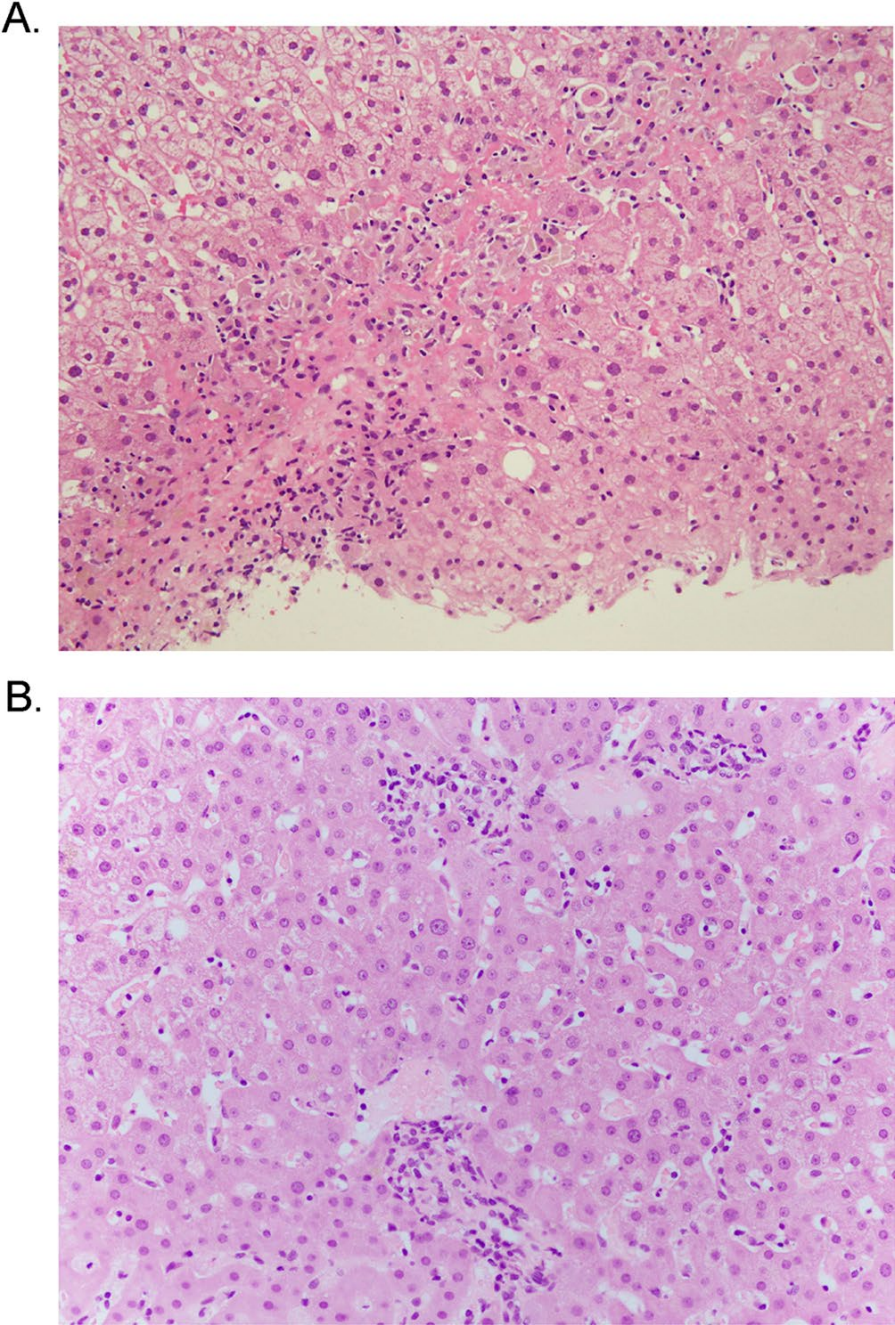
Hematoxylin and eosin staining of the liver biopsy in case 17 (**A**). Panlobular hepatitis was seen in this patient. Macrogranulomatous changes were easily identified and were composed of massive necrosis with inflammatory infiltrates comprising activated lymphocytes and histiocytes. Severe liver cell injuries and regenerative changes were also seen (**A**). Hematoxylin and eosin staining of liver biopsy in case 13 (**B**). Mild lobular hepatitis with microgranulomas was seen in this patient. Microgranulomatous changes were composed of foci of inflammatory infiltrates comprising activated lymphocytes and histiocytes. Mild liver cell injuries and regenerative changes were also seen (**B**).

**Figure 3:**
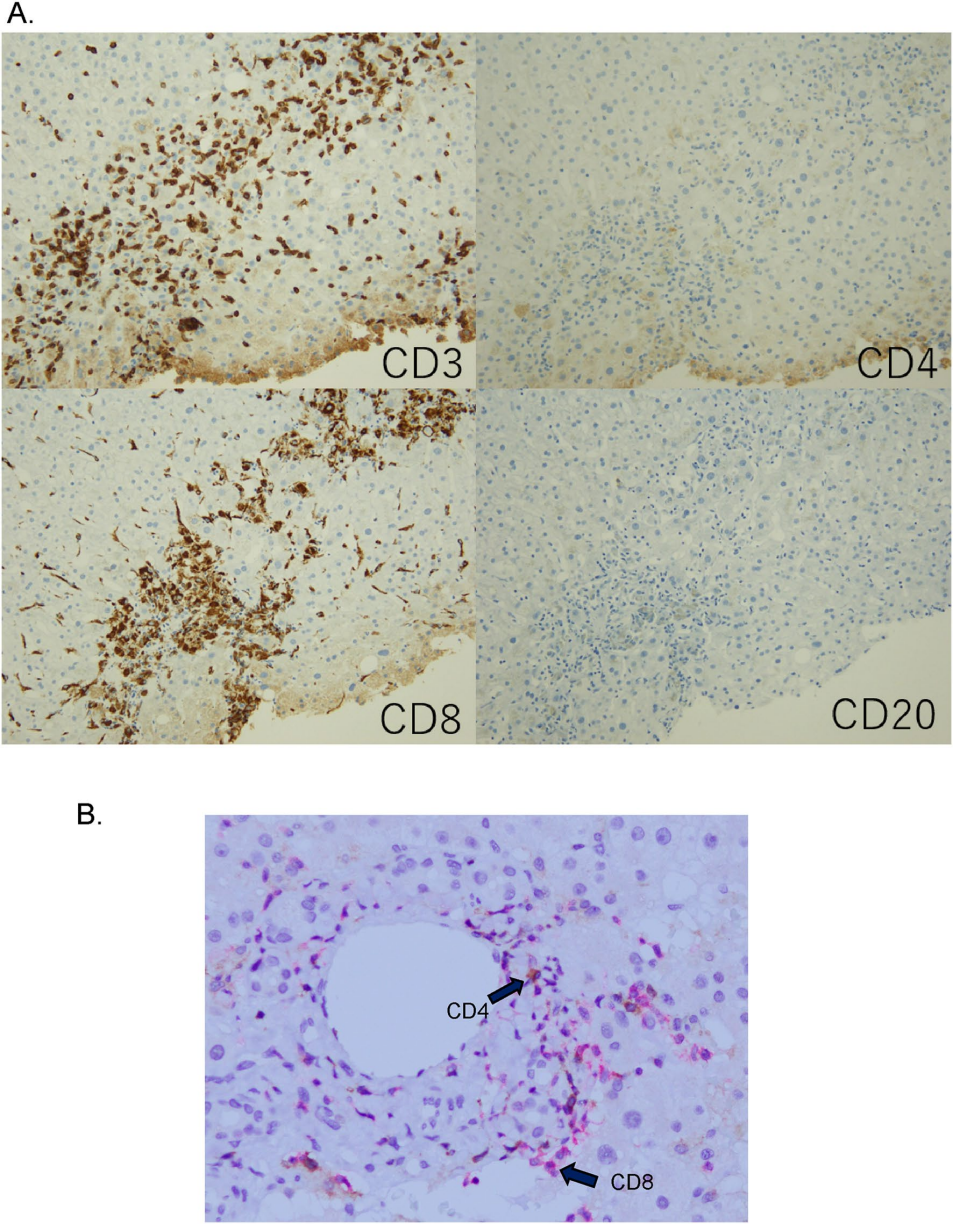
Immunohistochemical staining of the liver biopsy in case 17. Almost all infiltrating lymphocytes were CD3^+^/CD8^+^ cytotoxic T-cells (**A**). CD4^+^ T-cells, CD20^+^ B-cells and CD138^+^ plasma cells were rarely identified (**A**). Double-staining immunohistochemistry for CD4 (DAB: brown) and CD8 (fast-red: red) in case 17 (**B**). Red-stained lymphocytes were predominant, suggesting that most cells were CD8 positive lymphocytes (**B**).

Ten and 8 patients received liver biopsy after or during Nivolumab and Pembrolizumab treatment, respectively. The results of morphological and immunohistochemical analysis are summarized in Table 4. The degree of portal and lobular inflammation, fibrosis, and necrotic changes were significantly increased in patients with Grade3/4 patients compared to Grade1/2 patients (*p* < 0.05). Granulomatous changes, especially large granulomatous change with necrosis (Fig. 2A,B), were significantly increased in patients with ICIs patients compared with DILI and AIH patients (*p* < 0.05). The ratios of inflammatory cells CD4/CD8, and CD138/CD3 in ICIs (0.63 and 0.044) were significantly lower than those in AIH (1.89 and 0.21) or DILI (1.08 and 0.39) patients (*p* < 0.05, respectively) (Fig. 3A). Moreover, we carried out double-staining immunohistochemistry to visualize the proportion of CD4 and CD8 positive lymphocytes in case 17 (Fig. 3B). Red-stained lymphocytes were predominant, suggesting that most cells were CD8 positive lymphocytes. Steatosis, ballooning, and Mallory’s bodies were rarely identified in these patients.

## Discussion

The liver damage induced by treatment with iCIs has been widely recognized throughout the world. Various phenotypes of liver damage are induced by treatment with iCIs^11–13^. Most patients with iCIs-related liver damage revealed the phenotype of hepatocyte injury. We called this phenotype iCIs-related hepatitis. It is difficult to diagnose iCIs-related hepatitis by the clinical symptoms. Therefore, we diagnosed iCIs-related hepatitis by ALT level to avoid selection bias. This selection method might increase the frequency of iCIs-related hepatitis by PD-1 antibody. Some groups reported that iCIs treatment could induce cholangiocyte injury in a small number of patients^2,11^. It is difficult to confirm the representative phenotypes of liver damage induced by iCI treatment since various kinds of iCIs and the origins of the tumor could affect the pathogenesis of iCIs-related liver damage. Therefore, we focused on a single agent, anti-PD-1 therapy, with nivolumab or pembrolizumab for NSCLC to analyze the characteristics of hepatocyte injury. In our data, the median onset time points of iCIs-related hepatitis induced by the administration of nivolumab and pembrolizumab were 117 days and 114 days, respectively. Previously, another group reported treatment-related autoimmunity could occur within 5–15 weeks from the start of iCI treatment^14^. We found a positive rate of ANA in the nivolumab treatment group with hepatocyte injury that was significantly higher than that without hepatocyte injury. Moreover, we could detect significant differences among the pembrolizumab treatment groups even in such a small number of patients. In our institute, it was reported that preexisting antibodies and rheumatoid factors were more common among patients with irAEs^1^. In this study, AMA, IgG and IgM did not affect the incidence of liver damage. The pre-existence of some host factors including ANA might influence the induction of iCIs-related hepatitis.

Previously, some groups reported that NSCLC patients with various kinds of irAEs could have better OS and/or progression free survival than those without irAEs^15–18^. In this study, we could not detect significant differences in OS between patients with iCIs-related hepatitis and patients without iCIs-related hepatitis since we focused on iCIs-related hepatitis.

In addition to the topic of host factors that might induce iCIs-related hepatitis, the characteristics of the liver histology were important for the management of iCIs-related hepatitis^3–5,19–21^. Granulomatous changes, especially large granulomatous changes with necrosis, were significantly increased in patients with ICIs-related hepatitis compared with DILI and AIH patients in this study. Moreover, we found that the ratios of inflammatory cells CD4/CD8, and CD138/CD3 in ICIs were significantly lower than those in AIH or DILI patients. These data indicated that iCIs-related hepatitis might be related to CD8^+^ T cells. Another group reported that iCIs-related hepatitis characterized by predominant infiltration of CD8^+^ T cells in addition to regulatory T cells (Tregs) was seen in the liver biopsy specimens^22^. Dr. Okamura et al. analyzed how anti-PD-1 therapy could modify the cellular phenotypes of CD8^+^ T cells to destroy tumors and damage self-tissues^23^. They indicated that shifts in the cluster composition of autoreactive CD8^+^ T cells markedly reflected the severity of autoimmunity. Previously, another group reported that granulomatous hepatitis was observed in liver with grade ≥ 3 hepatitis caused by anti-PD-1/PD-L1 or CTLA-4 immunotherapies^19^. We were able to detect these characteristics of the liver histology when we carried out liver biopsy at the time of the G1 ALT elevation (5 patients / 7 patients). This finding could be helpful for managing patients with anti-PD1 treatment. It is difficult to decide on the discontinuation of PD-1 antibody and the use of steroid since such decisions might affect the survival of the patients.

ICIs-related hepatitis could be critical for patients of hepatocellular carcinoma (HCC) since many HCC patients could have limited liver function due to the liver cirrhosis. Previously, we reported that various kinds of factors including hepatitis B virus, hepatitis C virus, and the HCC microenvironment might affect the immune pathogenesis of liver diseases^24–30^. In this study, we included 3 HBsAg positive inactive carrier. Recently, it has been reported that iCIs treatment might induce hepatitis in the HBsAg positive patients^31–33^. It has been reported that anti-PD-1 blockade with nivolumab with and without therapeutic vaccination could cause a decline in HBsAg by inducing HBsAg-specific T cells in some patients^34^. In contrast, the change of immune function, especially, an alteration of CD8^+^ T cells and Tregs function by the use of iCIs might induce re-activation of HBV^35,36^. We should distinguish between the re-activation of HBV and iCIs-related hepatitis. In this study, we did not have experience about the re-activation of HBV during iCIs treatment. Therefore, it has been difficult to determine the characteristics of iCIs-related hepatitis by analyzing HCC patients treated with iCIs, so far. We should analyze HCC patients with various background diseases case by case in the near future.

In conclusion, we demonstrated that pre-existing ANA and a characteristic liver histology including CD8^+^ cells dominancy and granulomatous hepatitis could be biomarkers for the diagnosis of iCIs-related hepatitis in NSCLC with anti-PD-1 therapy. We should analyze the characteristics of iCIs-related hepatitis in various kinds of malignancies, and iCIs and combination therapy separately to understand the precise pathogenesis of iCIs-related hepatitis induced by specific treatment regimens of iCIs.
